# Supplementary figures and images for: ECOD: Classification of domains in AFDB Swiss-Prot structure predictions
Source: PLoS Comput Biol. 2026 Mar 30;22(3):e1013431. doi: 10.1371/journal.pcbi.1013431 (PMC13048469; doi:10.1371/journal.pcbi.1013431)

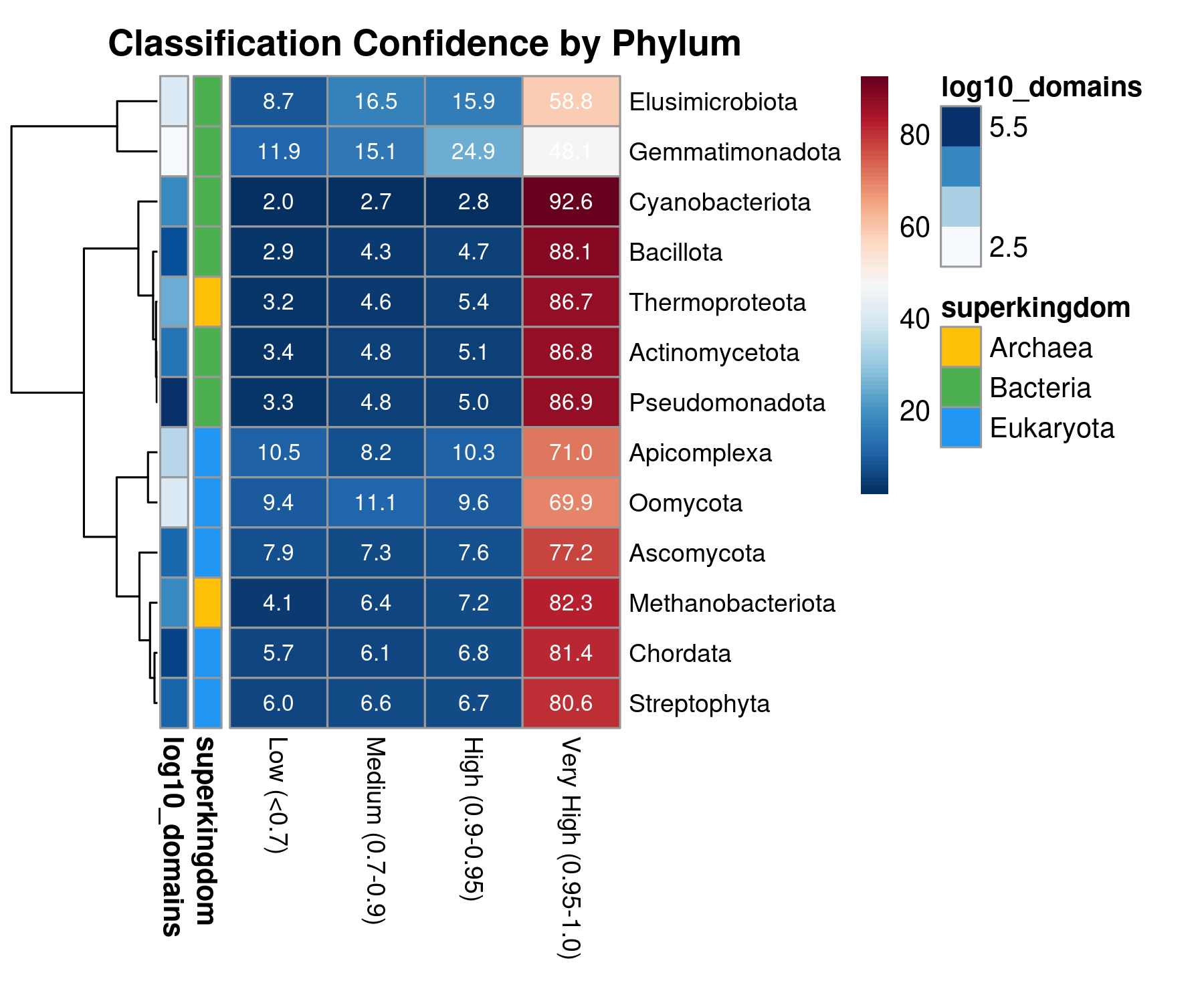

Supplement: S1 Fig — Heatmap showing classification confidence distribution across representative phyla, grouped into confidence bins, with hierarchical clustering and annotations for superkingdom and domain count. (TIFF) [file pcbi.1013431.s006.tiff]
