# Supplementary material for: ECOD: Classification of domains in AFDB Swiss-Prot structure predictions
Source: PLoS Comput Biol. 2026 Mar 30;22(3):e1013431. doi: 10.1371/journal.pcbi.1013431 (PMC13048469; doi:10.1371/journal.pcbi.1013431)
Supplement: Attachment — Submitted filename: Response to Reviewers for SwissProt.docx [file pcbi.1013431.s008.docx]

**Reviewers' comments:**

Reviewer's Responses to Questions

**Comments to the Authors:
Please note here if the review is uploaded as an attachment.**

*Reviewer #1: The manuscript by Schaeffer et al., "ECOD: Classification of domains in AFDB Swiss-Prot structure predictions" applies a previously developed DPAM pipeline to classify the protein domains from the AF-predicted structures. The manuscript substantially expands the coverage of classified domains by including over 500,000 structures from Swiss-Prot.
Furthermore, the submission substantially expands the phylogenetic coverage of species for which the domains have been annotated, going far beyond the 48 model organisms previously annotated by ECOD.
Expanding ECOD with predicted structures is overall an important work, but the manuscript in the current form doesn’t fully reflect it. There are several major issues that the authors need to address before the manuscript becomes suitable for publication in PLoS Computational Biology:*

1. With expanded phylogeny, ECOD should now offer an advanced phylogenetic browser/filtering, i.e., the ability to select, compare, or exclude domains across one or multiple taxonomic groups.

We agree that taxonomic filtering is an important capability given the expanded phylogenetic coverage. We have implemented an Advanced Taxonomic Search feature, currently available our main ECOD page (http://prodata.swmed.edu/ecod/af2_pdb/)-

The new functionality allows users to:

- Select one or more superkingdoms (Bacteria, Archaea, Eukaryota, Viruses) via multi-select checkboxes

- Apply cascading taxonomic filters at Phylum, Class, Order, Family, and Genus levels

- Combine taxonomic constraints with keyword and ECOD hierarchy searches

- View domain counts per taxonomic group to guide exploration

**This feature is enabled by taxonomic annotations now available for all SwissProt-derived domains (14,615 distinct taxonomy records across 2.7M domains in the test database).**
2. "4 superkingdoms" (line 93). The Consensus Statement by the International Committee on Taxonomy of Viruses indicates that "the current view [is] that viruses have multiple origins (polyphyly) and that their diversity cannot be represented by a single virosphere-wide tree" (doi: 10.1038/s41564-020-0709-x). Therefore, grouping viruses into a single fourth superkingdom is not supported. In line with this view, the NCBI changed the naming scheme**,** replacing "superkingdom" with "Acellular root" for viruses (<https://ncbiinsights.ncbi.nlm.nih.gov/2025/02/27/new-ranks-ncbi-taxonomy/>). Please comply throughout the text with the current consensus.

We thank the reviewer for this correction. We have updated the text to refer to three superkingdoms and clarified that viruses were outside the scope of this study as well as the previous 48 proteomes study. As the reviewer notes, viral taxonomy follows a distinct framework that would require separate consideration.

3. "Initial analysis shows Swiss-Prot contains a significantly higher density of functional annotations compared to reference proteomes" (lines 81-82). This should say Swiss-Prot entries and UniProt entries from reference proteomes. Additionally, is this initial analysis part of your manuscript? This is not clear; otherwise, please provide a reference.

We thank the reviewer for this observation. The original phrasing incorrectly implied novel analysis when this is a definitional property of Swiss-Prot. We have revised the text and added the UniProt reference.

4. The manuscript should provide a clear description of new domains identified in this study (maybe in the form of a table): which architectures and X groups expanded due to discoveries of new domains and which phylogenetic groups contributed to this expansion.

We thank the reviewer for this suggestion. We have substantially expanded our description of ECOD hierarchy expansion in the revised manuscript and supplementary materials. We have also updated all statistics to reflect the now-released ECOD v293, rather than the provisional version used in the original submission.

*Clarification of methodology*: The SwissProt DPAM pipeline assigns domains to the existing ECOD hierarchy—no new Architectures or X-groups are created through this automated process. Only new F-groups (families) can be instantiated to accommodate domains that match at the T-group level but do not fit existing families.

*Text additions to manuscript (Candidate domains section):*

We added a new paragraph describing hierarchy expansion:

"The SwissProt DPAM pipeline classifies domains into the existing ECOD hierarchy; no new architectures or X-groups are created through this automated process. All 20 ECOD architectures were expanded, with the largest contributions to the alpha/beta three-layered sandwiches (+169,515 domains) and the alpha+beta two-layer sandwiches (+114,278 domains). Taxonomically, bacterial proteomes dominated (76.9% of domains added), with notable exceptions from the Immunoglobulin-like beta-sandwiches (ECOD X: 11, 60.7% eukaryotic) and the repetitive alpha-hairpins (ECOD X: 109, 83.8% eukaryotic). ECOD v293 includes 260 new F-groups, of which 110 (42%) contain SwissProt domains; 29,720 domains (4.6%) remain classified at the T-group level (*.0 groups in ECOD) awaiting F-group assignment and Pfam classification."

*Supplementary Information tables added:*

| **SI Table** | **Description** |
| --- | --- |
| SI Table 1 | Architecture-level expansion (all 20 architectures with v292 baseline and SwissProt contribution) |
| SI Table 2 | X-group expansion (top 30 by SwissProt contribution) |
| SI Table 3 | Taxonomic attribution by X-group (% Bacteria/Eukaryota/Archaea for X-groups with ≥1,000 domains) |
| SI Table 4 | New F-groups summary by parent T-group |
| SI Table 5 | T-group only assignments (top 20 T-groups awaiting F-group assignment) |

*Zenodo repAlriository updates*:

We reorganized and expanded our Zenodo data repository to include:

- xgroup_expansion.csv — Complete X-group expansion statistics (2,348 X-groups)
- xgroup_taxonomy.csv — Taxonomic breakdown for all X-groups with SwissProt domains
- new_fgroups.csv — All 260 new F-groups in v293, with columns indicating SwissProt domain presence
- tgroup_only_domains.csv — Complete listing of 29,720 domains awaiting F-group assignment
- Updated accessioned_domains.csv — All 648,979 accessioned domains with F-group assignments and taxonomic attribution

*Key findings highlighted:*

- All 20 ECOD Architectures were expanded by SwissProt classification
- Bacterial proteomes dominate (76.9%), but two X-groups show strong eukaryotic representation: Immunoglobulin-like β-sandwiches (60.7% eukaryotic) and Repetitive α-hairpins (83.8% eukaryotic)
- SwissProt domains contributed to 42% of the new F-groups created in ECOD v293 (110 of 260)
- 29,720 domains (4.6%) remain at T-group level, representing candidates for future F-group creation

5. The authors state that "the propagation of functional annotation from experimentally characterized proteins and their domains to their homologous yet hypothetical or uncharacterized domains can lead to biological insights" (lines 54-56). Yes, but it can also lead to errors. While there is clearly value in functional annotation, please be specific and provide examples and references.

We thank the reviewer for this suggestion. We have added citations to specific examples where homology detection led to functional inference (Kinch & Grishin, 2002; Pei & Grishin, 2009) and expanded the text accordingly.

Minor comments:

6. The authors start this manuscript with the following sentence: "Protein domains are independent evolutionary units that convey function and fitness" (line 51). This appears to be an overstatement; please provide references showing that protein domains in general provide fitness or remove statements that are unspecific, vague, and unverifiable assumptions.

We have added citations supporting the characterization of domains as evolutionary units (Doolittle 1995, Bornberg-Bauer et al. 2005, Dohmen et al. 2020) and removed the claim regarding fitness.

7. Figure 1. Panel D: labels are cropped and inconsistent. The figure legend for panel D also needs revision as the names on the plot do not correspond to the names in the legend. In the plot we have "EC Annotations", "EC Proteins", and "Unique ECs"; in the legend we have "proteins with at least one EC number", "total EC annotations", and "unique EC identifiers".

We have revised Figure 1D: shortened facet labels to avoid clipping, corrected bar coloring, and updated the caption to define the intersection group (Int) and match facet terminology.

8.) Figure 2. Panel A: the y-axis title is cropped. Please fix the color codes: in Panel B, as per the figure legend, domain count is shown in blue and residue count in green. However, it is confusing that the label "Simple Topology" is shown in the same green as residue count bars, and the label "Good" is shown in the same blue as domain count bars. Are the four categories "Partial", "Simple Topology", "Low Confidence", and "Good" mutually exclusive? Please define.

We have corrected the cropped y-axis label in Panel A and revised the color scheme to eliminate confusion between count type (Domains/Residues) and classification categories. The four judge categories are mutually exclusive; each domain receives exactly one classification based on DPAM confidence, template coverage, and secondary structure content: good_domain (high-confidence match with ≥3 SSEs), partial_domain (high-confidence but incomplete coverage with ≥3 SSEs), low_confidence (uncertain assignment with ≥3 SSEs), or simple_topology (<3 SSEs).

9. Panel C appears to follow the same color code as panel B, but this is not specified; please clarify. The bars in panel D appear to follow the colors indicated by the labels in panel B; please clarify.

We have revised the figure caption to explicitly state that panels B, C, and D use consistent color schemes throughout.

10. Please provide a definition for "Simple Topology". The text states that "Simple topology domains (9.20% of domains but only 2.54% of residues) typically represent smaller structural units." Is this a or a definition?

We have added a formal definition of simple_topology to the Methods section. Simple topology domains are those containing fewer than three secondary structure elements (SSEs), where helices of at least 6 residues and strands of at least 3 residues each count as one SSE. These typically represent short peptides, minimal repeat units, or regions with sparse regular secondary structure.

11. Lines 263-269 and 290-297 describe the same results. Please avoid repetition.

We have removed the repetition from these lines.

Reviewer #2: This manuscript presents an extension of the Evolutionary Classification of Protein Domains (ECOD) to include AlphaFold Database (AFDB) structure predictions for all Swiss-Prot proteins. Using the Domain Parser for AlphaFold Models (DPAM) pipeline, the authors classify over 1 million domains derived from more than 540,000 protein structure predictions, achieving broad taxonomic and functional coverage. The study integrates structure-based domain classification with Swiss-Prot’s curated functional annotations and provides a comprehensive dataset deposited in Zenodo and the ECOD database.

Overall, this is a valuable resource because of the following strengths:
1. The work expands ECOD’s coverage into a major curated dataset (Swiss-Prot), representing an important addition to structural bioinformatics resources.
2. The authors describe a systematic and reproducible pipeline (DPAM + ECOD + Pfam integration), combining sequence, structure, and taxonomic analysis.
3. The reported high DPAM probability (mean 0.992) and extensive pLDDT validation underscore robust classification.
4. Data are openly available via Zenodo and ECOD.
5. The manuscript provides taxonomic, functional, and evolutionary insights—e.g., the discovery of >100,000 domains lacking Pfam mappings—highlighting areas for future functional annotation and structural validation.

However, there are still two issues for the authors to address:
• A more detailed analysis or discussion of the difference and similarity between ECOD extension with CATH’s use of AFDB can strengthen the work.

The relationship between ECOD/DPAM and CATH/TED classifications has been systematically examined in recent work comparing domain boundaries and fold assignments between the two frameworks (Pei et al. 2025a, 2025b). These studies found that a substantial fraction of TED candidate novel-fold domains represent distant homologs of existing ECOD entries, and that the two approaches differ primarily in domain boundary definitions rather than fundamental disagreements about fold space. The SwissProt proteins analyzed here are predominantly well-characterized with established domain architectures, distinct from the marginal cases examined in those comparative studies.

• Typographical errors (e.g., “Classificatioxn”) should be corrected.

We have corrected this and other typographical errors.
