# Supplementary material for: ECOD: Classification of domains in AFDB Swiss-Prot structure predictions
Source: PLoS Comput Biol. 2026 Mar 30;22(3):e1013431. doi: 10.1371/journal.pcbi.1013431 (PMC13048469; doi:10.1371/journal.pcbi.1013431)
Supplement: Attachment — Submitted filename: Response to Reviewers R2 - ECOD SwissProt.docx [file pcbi.1013431.s009.docx]

Response to Reviewers R2

We thank the reviewers for their constructive comments. We have addressed each point and describe the corresponding changes to the manuscript below.

**Reviewer #1**

“The revised version of the manuscript essentially addressed all the concerns raised in the original submission. I recommend this manuscript for publication after a couple of minor glitches are fixed.”

We thank Reviewer #1 for the positive assessment and have addressed both points below:

“In the responses, the authors stated that the advanced taxonomic search is available from the main ECOD page <http://prodata.swmed.edu/ecod/af2_pdb/>. It appears that this useful feature is available from <http://prodata.swmed.edu/ecod/af2_pdb/search/advanced>. Please make it available at the front page or provide an explicit link in the manuscript. It may also be beneficial to deploy an SSL connection to the server using Certbot or other services.”

We have modified the webpage to foreground the new advanced search and added a news items to reflect the release of these new capabilities. We wholeheartedly agree with the reviewer regarding the SSL cert. This is an ongoing saga and the external request from the reviewer provides us with more evidence for relevant parties that this is a necessary improvement. We hope that HTTPS will be deployed along with our new webpage as part of our 2026 rollout

“While the 4 superkingdom issue has been addressed in the revised version, line 93 still states "with domains spanning 4 superkingdoms, 98 phyla, and 10,254 species". Please fix.”

Fixed. The text now reads "with domains spanning 3 superkingdoms, 91 phyla, and 10,254 species." We have verified that no other instances of "4 superkingdoms" remain in the manuscript. The phyla count was also corrected from 98 to 91 to reflect domain-bearing species only.

**Reviewer #3**

We thank Reviewer #3 for the detailed and constructive review. We have made revisions to address each point.

**Major Point #1**

“Fixed. The text now reads "with domains spanning 3 superkingdoms, 91 phyla, and 10,254 species." We have verified that no other instances of "4 superkingdoms" remain in the manuscript. The phyla count was also corrected from 98 to 91 to reflect domain-bearing species only.”

We agree that the original characterization of unmapped domains as “novel evolutionary groups” was overstated. We have substantially revised the Pfam mapping analysis to precisely characterize the sources of Pfam/ECOD discrepancy.

***New analysis and findings:***

Of the 52,644 unmapped well-assigned domains, we find that:

- **85.7% (45,129)** match the same ECOD structural references as Pfam-mapped domains in their topology group. These are sequence-divergent members of known structural families where an appropriate Pfam HMM exists but cannot detect these particular sequences (a *sensitivity gap* between structure- and sequence-based methods). These domains are shorter (median 105 vs. 149 residues) and more frequently discontinuous (21.5% vs. 13.7%) than mapped domains.
- **14.3% (7,515)** match ECOD structural references not used by any Pfam-mapped domain -- structurally classified sub-groups within known topology groups that lack corresponding sequence profiles (*underspecified sub-groups*).
- Only **1%** of unmapped domains reside in topology groups with no Pfam representation at all.

To validate this interpretation, we scanned these unmapped domains against Pfam releases from v37.4 through v38.2 (this manuscript was calculated against v38). Since the original writing of this draft we communicated these unmapped domains to the Pfam curators. Pfam v38.2 captures 16.7% (8,797) of previously unmapped domains, with 91% of these captured domains being attributed to families created subsequent to our original analysis.

***Changes to manuscript:***

- Abstract and Introduction: We replaced “novel evolutionary groups” with more precise language.
- Results: We expanded the Pfam mapping section with the results described above
- Discussion: reframed the unmapped domains as demonstrating the collaboration between structural and sequence classifications
- Methods: added a paragraph describing the Pfam gap characterization and the temporal scan methodology

**Major Point 2**

“In lines 325-326, what does the fact that there are many unannotated but with high-quality ordered structures mean? Are they truly new domains, structures, that were absent in experimental DBs? Or is it due to disparity in sequence similarity and structure similarity?”

The expanded analysis described above (Major Point 1) directly addresses this question. The high-confidence unmapped domains are predominantly sequence-divergent members of known structural families (85.7%), not structurally novel entities. Their high pLDDT scores confirm well-folded structures, while their lack of Pfam annotation reflects the detection sensitivity boundary between structural and sequence-based methods. We have revised the relevant text to make this distinction explicit, noting that 98.9% of unmapped well-assigned domains belong to ECOD topology groups where other domains successfully map to Pfam.

**Major Point 3**

**“**Although ECOD was published some time ago, a proper introduction of the hierarchy, organization, and the rationale of the design the ECOD classification framework should be provided. The meanings of the H-, T-, and X-level annotations should be briefly explained to make the results accessible to a broader audience.**”**

We have added a paragraph to the Introduction describing the ECOD hierarchy:

**Major Point 4**

**“**Even with rather low similarity, 40%, the number of singletons is almost half of the entire clusters. Does this mean that the current parsing scheme is too strict or sensitive to small changes in structures? Authors should provide more explanation and discussion on these large number of singletons.**”**

We have expanded the clustering results section to clarify this important distinction and provide a thorough characterization of singletons.

The key insight is that while singletons comprise 43.5% of *clusters* at 40% identity, they represent only 6.7% of all domains (69,908 of 1,032,610). The remaining 93.3% of domains successfully cluster with at least one other domain. Among well-assigned domains, 97.9% of singletons at 40% are also singletons at 70%, indicating that these are genuine unique sequences rather than borderline threshold cases.

**Major Point 5**

“Figure 2D is not referenced in the text and the color codes of Figure 2D are not presented in the legend.”

We have added a reference to Figure 2D in the results text and added a color legend for the judge categories (Good, Simple Topology, Partial, Low Confidence) to the Figure 2 legend.

**Major Point 6**

**“**Figure 3 is small and difficult to interpret.**”**

We have redesigned Figure 3 as a 2-panel figure for a 2-column journal layout. The original 3-panel layout compressed each panel excessively. Panel A (top 10 H-groups by domain count, stacked by superkingdom) and Panel B (pLDDT violin plots comparing Swiss-Prot and 48P datasets) are now displayed side-by-side with increased font sizes and spacing. The former Panel C (phylum-level confidence distribution heatmap) has been moved to S6 Fig as a standalone supplementary figure, where it can be displayed at full size without competing for space.

**Minor Point 1**

**“**In line 88, "Swiss-Prot/UniProtKB" should be corrected to "UniProtKB/Swiss-Prot.**”**

Corrected throughout the manuscript

**Minor Point 2**

**“**In line 313, pfam -> Pfam**”**

Corrected. We verified the correct capitalization throughout the manuscript
